# Primary Hepatoid Adenocarcinoma of Gallbladder With MB21D2/GALNT12/ARID2 Mutations: A Case Report

**DOI:** 10.3389/fendo.2021.791153

**Published:** 2022-01-03

**Authors:** Zhenyu Li, Qingming Jiang, Xinyu Chen, Yu Xiao, Jue Xiao

**Affiliations:** ^1^ Department of Pathology, Chongqing University Cancer Hospital, Chongqing, China; ^2^ Department of Pathology, Liangping People’s Hospital, Chongqing, China

**Keywords:** hepatoid adenocarcinoma, gallbladder, TMB, case report, immunocytochemistry

## Abstract

**Background:**

Primary hepatoid adenocarcinoma of the gallbladder is a relatively rare type of extrahepatic adenocarcinoma. The genetic changes involved in this type of adenocarcinoma were unexplained so far. We reported a rare case of primary hepatoid adenocarcinoma of gallbladder with Mab-21 domain containing 2 (MB21D2), polypeptide N-acetylgalactosaminyltransferase 12 (GALNT12), and AT-rich interaction domain 2 (ARID2) mutations, which was confirmed after surgical resection pathologically.

**Case Summary:**

A 69-year-old female with distention of hypogastrium and constipation received enema treatment, but ineffectively. No abnormalities were found on relevant physical examination. Then, the CT and MRI demonstrated a 3.3–4-cm soft tissue mass shadow in the neck of the gallbladder. The primary lesions consisted of two components: high-grade intraepithelial neoplasia of glands and hepatoid glands microscopically after laparoscope cholecystectomy. Immunohistochemical staining showed the sameness and difference of the two areas. Furthermore, tumor mutational burden (TMB) shows that the MB21D2, GALNT12, and ARID2 genes were mutated.

**Conclusion:**

This is the first report of primary hepatoid adenocarcinoma of the gallbladder with MB21D2, GALNT12, and ARID2 mutations. This will provide a theoretical basis for genetic changes in rare tumors.

## Introduction

Hepatoid adenocarcinoma (HAC) is a very rare extrahepatic tumor which originates in the gastrointestinal tract and morphologically and functionally resembles hepatocellular carcinoma (HCC) (1). HAC could arise in the lung (2), stomach (3), uterine cervix (4), colon (5), bladder cancers (6), etc. Primary HAC of the gallbladder is a relatively rare type of extrahepatic adenocarcinoma. To our knowledge, only 27 relevant studies about HAC of the gallbladder have been published in the National Center for Biotechnology Information (NCBI) database so far, and these mostly focus on pathologic findings since first authoritatively introduced in 1995 (7). Genetic detection is not only crucial to the diagnosis for malignant tumors but also used to assess the prognosis in gallbladder cancers (8, 9). No research on genetic changes in HAC of the gallbladder has been reported so far. Here, we used a tumor mutational burden (TMB) of 688 genes detected by MGISEQ-2000 of Beijing Genomics Institute as a support to explore the genetic changes after histology and immunohistochemistry and reported a unique primary HAC of the gallbladder with MB21D2, GALNT12, and ARID2 mutations.

## Case Description

This is a Chinese case of a 69-year-old female who noticed distention of the hypogastrium accompanied by constipation 20 days ago. No pain, nausea, vomiting, fever, oppression in the chest, jaundice, and bloody stool were found during the period, and she went to the local hospital and received enema treatment. However, distention of the hypogastrium still existed. There were no liver palms, spider-burst, superficial lymph node enlargement, tenderness, rebound tenderness, masses, fluid thrill, shifting dullness, hepatojugular reflux, and pathological reflex on relevant physical examination. The patient denied familial genetic, alcohol abuse, hepatitis, hepatic cirrhosis, psychosocial and exposure to radiation, and toxin history. Furthermore, she was examined in line with the care checklist, as shown in **Supplementary Figure 1**.

Laboratory tests of the tumor biomarkers (alpha-fetoprotein (AFP), CEA, CA125, CA199, CA153), liver function (ALT, AST, TP, ALB, GLB, TBIL, DBIL, IBIL), and lipid index (TG, TC, HDL-C, LDL-C) of serum were normal.

**Figure 1:**
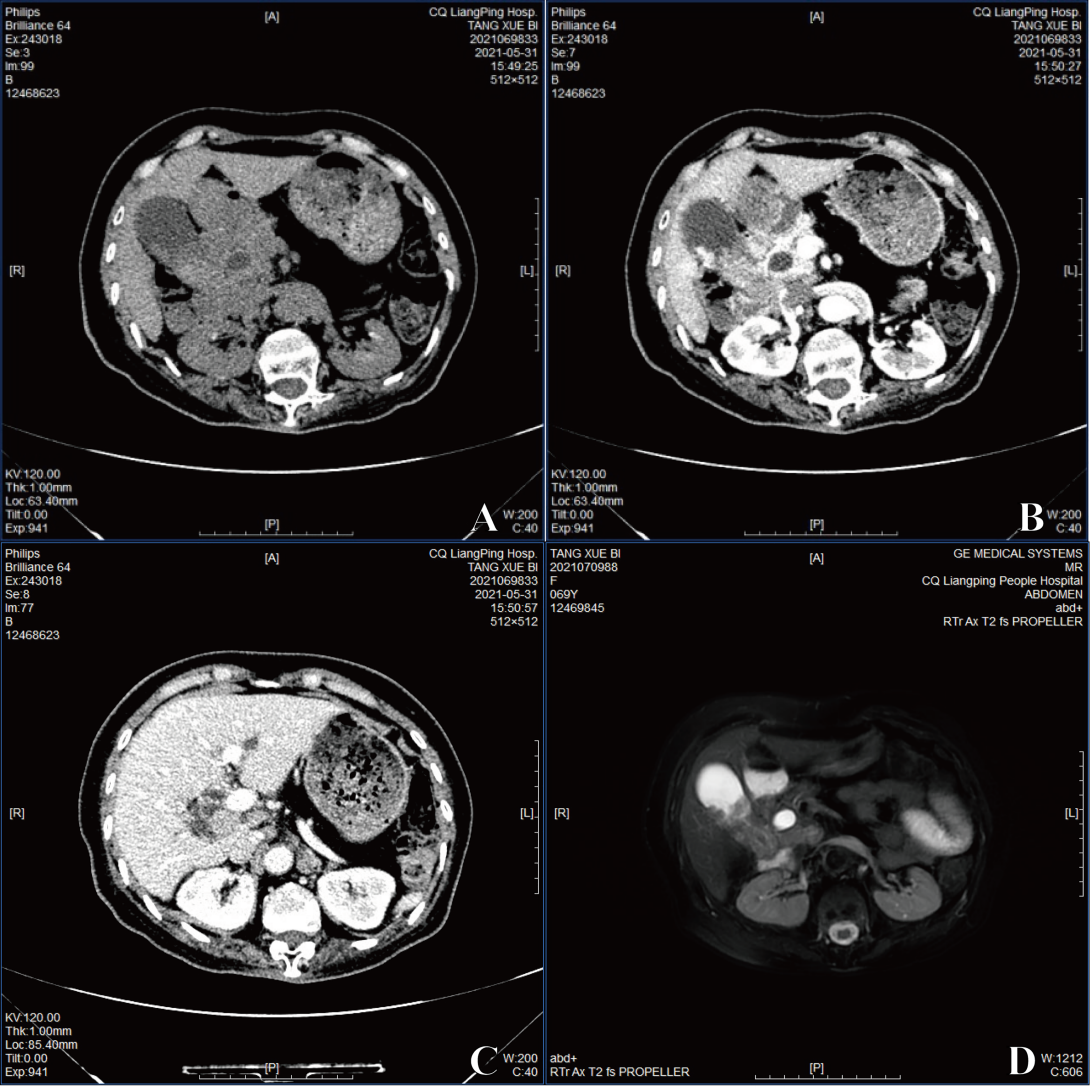
**(A)** The image exhibited with CT. **(B)** The image of enhanced CT. **(C)** Porta hepatis lymph node enlargement by enhanced CT. **(D)** The image of MRI.

She was admitted and underwent abdominal computed tomography (CT), which demonstrated a 3.3–4-cm soft tissue mass shadow in the neck of the gallbladder which was significantly enhanced on enhanced scan with an unclear boundary to the duodenum and head of pancreas (**Figures 1A, B** **)**. Unclear cystic duct, dilation of the common bile duct, and bile ducts inside and outside the liver were shown. No dilation of the pancreatic duct or definite abnormality of the liver was detected. Porta hepatis lymph node enlargement was also observed (**Figure 1C**). Magnetic resonance imaging (MRI) also showed gallbladder neck mass (**Figure 1D**). Therefore, a gallbladder carcinoma was suspected and laparoscope cholecystectomy was performed.

**Figure 2:**
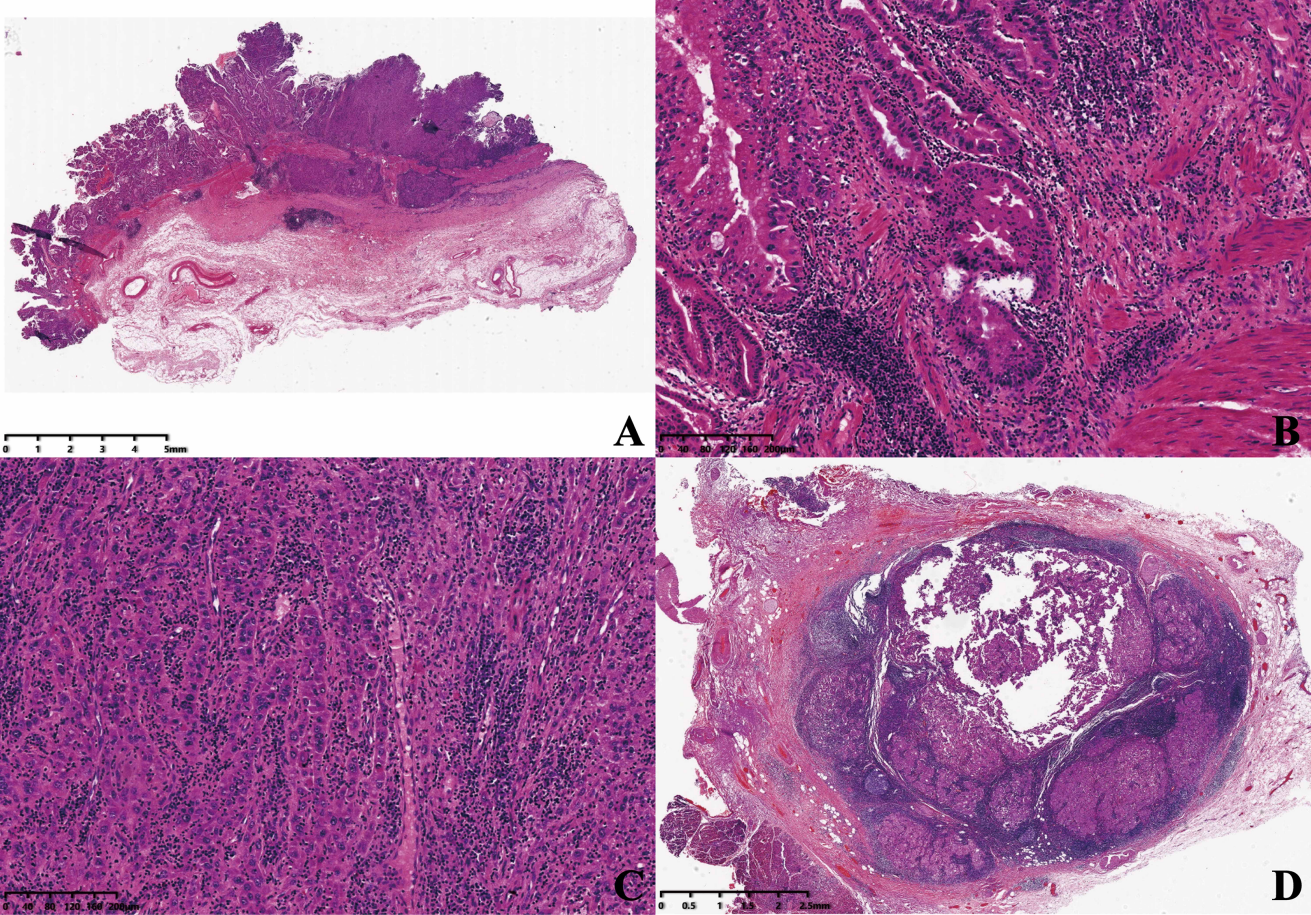
**(A)** The mass was shown microscopically (×5). **(B)** High-grade intraepithelial neoplasia of glands (×100). **(C)** Hepatoid glands (×100). **(D)** Porta hepatis lymph node (×10).

The mass was about 3.8 × 3.5 × 1 cm^3^ in size on the gallbladder wall (**Figure 2A**). The primary lesions consisted of two components: high-grade intraepithelial neoplasia of glands (**Figure 2B**) and hepatoid glands (**Figure 2C**). The tumor cells were cuboidal or polygonal with abundant eosinophilic granular hepatocyte-like neoplastic cells, and the nucleus was large and ovoid; moreover, one to two nucleoli were also shown in the area of the hepatoid component. The lymph node around the gallbladder had been invaded by the hepatoid tumor cells (**Figure 2D**).

**Figure 3:**
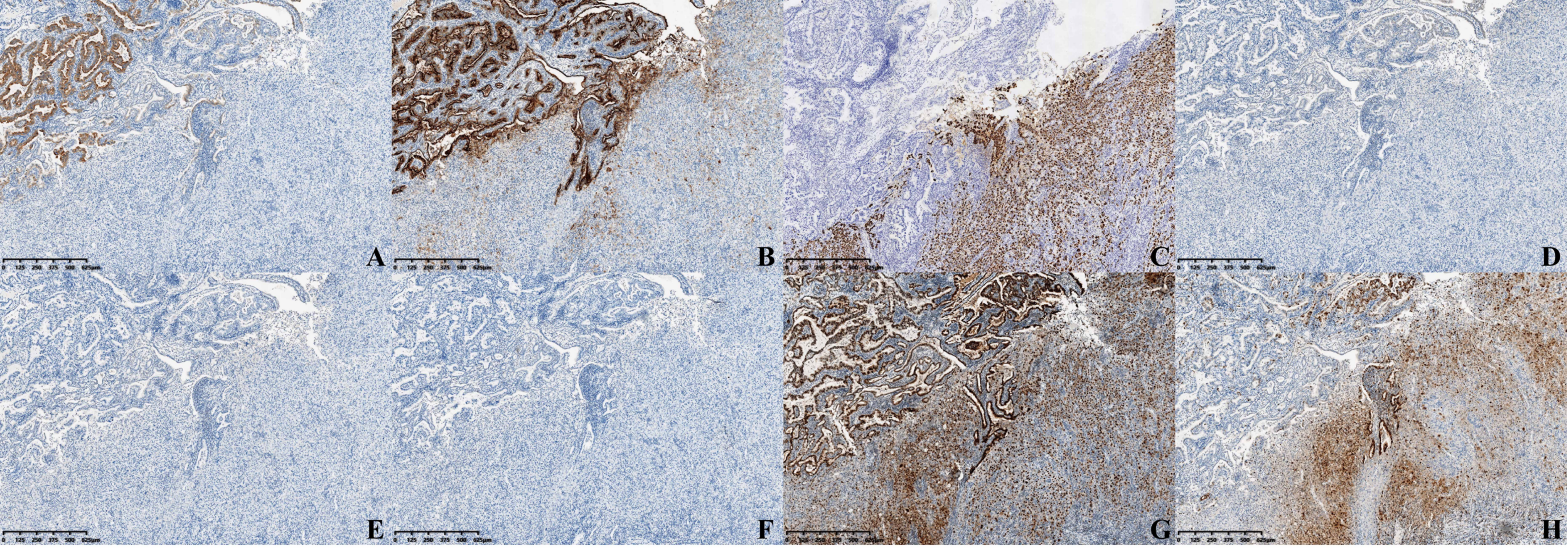
Immunohistochemical staining (×40) of **(A)** Glypican-3, **(B)** MUC-1, **(C)** Hep par-1, **(D)** AFP, **(E)** Arginase-1, **(F)** Sall-4, **(G)** P53, and **(H)** Ki-67.

Immunohistochemical staining shows positiveness of glypican-3 and MUC-1 of high-grade intraepithelial neoplasia of glands, but negativeness or weak positiveness in the hepatoid glands (**Figures 3A, B** **)**. Hepatocyte paraffin 1 (Hep par-1) showed contrasting results (**Figure 3C**). AFP, arginase-1, and Sall-4 were all negative (**Figures 3D–F**), and P53 was strongly positive (**Figure 3G**). The Ki-67 index in hepatoid glands was much stronger than that of other areas (**Figure 3H**). Besides, positiveness of CK-pan, CK7, CK8, CK18, and CK19 and negativeness of CK20 were also detected.

TMB of 688 genes detected by MGISEQ-2000 of Beijing Genomics Institute (BGI, Shenzhen, China) was used in the case. The MB21D2 gene showed Exon 2E p.Q311E (c.931C > G) mutation, and abundance of mutation was 10.5%. The GALNT12 gene revealed Exon 1 p.L133Q (c.338T > A) mutation, and abundance of mutation was 13.56%. The ARID2 gene indicated Exon 10 p.L381V (c.1141C > G) mutation, and abundance of mutation was 11.47% (**Table 1**). No microsatellite instability (MSI) and germline mutation were detected. Finally, we made the diagnosis of primary hepatoid adenocarcinoma of the gallbladder with MB21D2, GALNT12, and ARID2 mutations.

**Table 1:** Somatic cell variation bt TMB.

Gene Name	Detection Result	Gene Subregion	Transcript ID	Abundance of Mutation	Variation Level
MB21D2	Exon 2E	p.Q311E (c.931C > G)	NM_178496.3	10.5%	II
GALNT12	Exon 1	p.L133Q (c.338T > A)	NM_024642.4	13.56%	III
ARID2	Exon 10	p.L381V (c.1141C > G)	NM_152641.2	11.47%	III

II, potential clinical significance; III, unclear clinical significance.

The patient remained under careful observation by radiological and ultrasonic examination, and there was no visceral metastasis in the 5-month follow-up. The patient got an appropriate perspective including the assessment and the episode of care in every month, such as ultrasound examination, rehabilitation training, dietary guidance, regular physical examination, and mental status assessment. The patient healed well, and the distention of hypogastrium disappeared. No adverse or unanticipated events and symptoms in physical examination happened during the period.

## Discussion

HAC is defined as a relatively rare tumor consisting of adenocarcinoma admixed with foci of the tumor resembling mature and neoplastic hepatocyte which is proposed as a specific subtype with poor prognosis (10). In our case, the primary lesions consisted of high-grade intraepithelial neoplasia of glands and hepatoid glands. Besides, the lymph node around the gallbladder had been invaded by the hepatoid tumor cells. No special differentiation, such as squamous differentiation of the tumor cells, was detected as in the previous report (11). The morphology of the invaded tumor cells in the lymph node was hepatoid glands, so we first preferred the diagnosis of HAC of the gallbladder.

In this case, CT and MRI revealed that the tumor spanned beyond the neck of the gallbladder. The patient was tested with a normal level of AFP and had no history of alcohol abuse, hepatitis, or hepatic cirrhosis, and thus, prior to liver function, radiologic, and surgery findings, we considered it possible that the origin of this tumor was the gallbladder. Patients with HAC always have markedly elevated circulating levels of AFP (7), although normal levels of AFP have also been reported (11–13). In our case, the serum AFP was also associated with a normal level, and the immunohistochemical staining of AFP was negative at the same time. It reminded that AFP positivity is not necessarily diagnostic of HAC, because of the uncertain AFP overproduction.

Immunohistochemical staining results also supported the HAC. In our case, in the hepatoid gland area, we found negativeness of glypican-3 and Sall-4. A report indicated that glypican-3 and Sall-4 were approximately 87.5% and 93.8% positive in HAC, respectively (14), which meant that glypican-3 and Sall-4 could be negative or dedifferentiation had occurred in the hepatoid glands. This was the strange immunophenotype in this case. Arginase-1 is a novel sensitive and specific marker for HCC, but not for HAC (15), so it was negative in our case. Mucin core protein 1 (MUC1) is expressed in the benign and malignant lesions of the gallbladder and closely related to the carcinogenesis of gallbladder adenocarcinoma (16). In our case, MUC-1 was positive in high-grade intraepithelial neoplasia of glands, but weakly positive in hepatoid glands. Thus, the transitional zone of two components of tumor cells was morphological, so we were more certain that the origin of this tumor was the gallbladder.

The ErbB signaling pathway was related to somatic mutational landscape in gallbladder carcinoma including TP53, KRAS, and ERBB3 (17). However, studies available in literature do not clearly define the molecular genetic mechanisms involved in the pathogenesis of gallbladder carcinoma, including HAC (18). However, in this case, we found the mutations of MB21D2, GALNT12, and ARID2 genes.

MB21D2, as a novel cancer gene, which belongs to an intracellular cadherin binder, is found to harbor Q311E recurrent mutation and to be overexpressed in head and neck cancer and involved in cellular processes, including cell survival, proliferation, and migration (19). MB21D2 is also frequently mutated in lung cancers involved in tumor suppression and cancer resistance (20). MB21D2 mutation is not found in HAC of the gallbladder ever. Unfortunately, we do not know how the MB21D2 mutation will affect this patient.

GALNT12 is a strong candidate colorectal cancer-susceptibility gene given previous linkage and association studies, inactivating somatic and germline alleles in colorectal cancer patients (21). GALNT12 was also associated with follicular lymphoma, endometrioid endometrial carcinoma, and B-cell non-Hodgkin’s lymphoma (22–24). The involvement of GALNT12 in HAC of gallbladder is also not clear yet and is needed for further research.

ARID2 contributes to disruption of the DNA repair process, resulting in susceptibility to carcinogens and potential hypermutation in HCC (25), especially in the Asian race (26). ARID2 mutants with a disrupted C2H2 domain lose the metastasis suppressor function, exhibiting a positive association with HCC metastasis and poor prognosis (27). ARID2 defines some of the core-deregulated pathways in HCC and will be an ideal biomarker for specific therapeutic decisions, which is an unmet medical need in this field (28). The morphology and function of HAC are similar to that of HCC (29, 30), ARID2 mutation may provide a theoretical basis for specific therapeutic approaches of HAC.

In this case, the challenges of the diagnosis included the rare site of tumor, no obvious symptoms in physical examination, variation of the immune phenotype by immunohistochemical staining, etc. We needed to combine sufficient clinical information, imaging, histological morphology, and multiple immunohistochemical indicators to make the final diagnosis.

It is worth noting that the lymph node around the gallbladder had been invaded, which reminded that distant metastasis may occur again in the patient, so we recommend a close follow-up on her. The strength of this case was the specific and rare genetic changes which were firstly reported; unfortunately, we cannot find specific targeted therapeutics for further treatment, and we did not use first-generation sequencing of MB21D2, GALNT12, and ARID2 genes to verify the TMB outcome, which were the limitations of this case.

Besides, the patient remained under careful observation, got an appropriate perspective in every month, and received no adverse and unanticipated events after treatment. She felt satisfied and appreciated for the treatment plan, prognosis, and follow-up observation and, furthermore, would continue to be followed up as prescribed. We will continue to monitor the patient’s situation.

In summary, we presented an unusual and rare case of HAC in the gallbladder with MB21D2, GALNT12, and ARID2 mutations, which was first reported. Although HAC has characteristic histological features, awareness is important for its diagnosis and prognosis. Therefore, this case will provide a theoretical basis for genetic changes in rare tumors.

## Data Availability Statement

The patient provided informed consent for the publication of this report and any accompanying images.

## Ethics Statement

Informed consent was obtained in this case, and protocols were approved by the Ethics approval of Chongqing University Cancer Hospital and Liangping People's Hospital.

## Author Contributions

ZL contributed to the acquisition, analysis, and interpretation of patient data and the drafting of the manuscript. QJ contributed to the acquisition of the CT and MRI examination data. XC contributed to the HE, immunohistochemistry, and molecular pathological methods. JX and YX gave the final approval of the report. All authors contributed to the article and approved the submitted version.

## Conflict of Interest

The authors declare that the research was conducted in the absence of any commercial or financial relationships that could be construed as a potential conflict of interest.

## Publisher’s Note

All claims expressed in this article are solely those of the authors and do not necessarily represent those of their affiliated organizations, or those of the publisher, the editors and the reviewers. Any product that may be evaluated in this article, or claim that may be made by its manufacturer, is not guaranteed or endorsed by the publisher.

## Supplementary Material

Supplementary Figure 1 Timeline with relevant data from the episode of care.

## Abbreviations

HAC, hepatoid adenocarcinoma; HCC, hepatocellular carcinoma; NCBI, National Center for Biotechnology Information; CT, computed tomography; MRI, magnetic resonance imaging; MB21D2, Mab-21 domain containing 2; GALNT12, polypeptide N-acetylgalactosaminyltransferase 12; ARID2, AT-rich interaction domain 2; AFP, alpha-fetoprotein; MUC1: Mucin core proteins 1; Hep par-1, hepatocyte paraffin 1; CK, cytokeratin; TMB, tumor mutational burden; MSI, microsatellite instability.
