# Supplementary figures and images for: Primary Hepatoid Adenocarcinoma of Gallbladder With MB21D2/GALNT12/ARID2 Mutations: A Case Report
Source: Front Endocrinol (Lausanne). 2022 Jan 3;12:791153. doi: 10.3389/fendo.2021.791153 (PMC8761856; doi:10.3389/fendo.2021.791153)

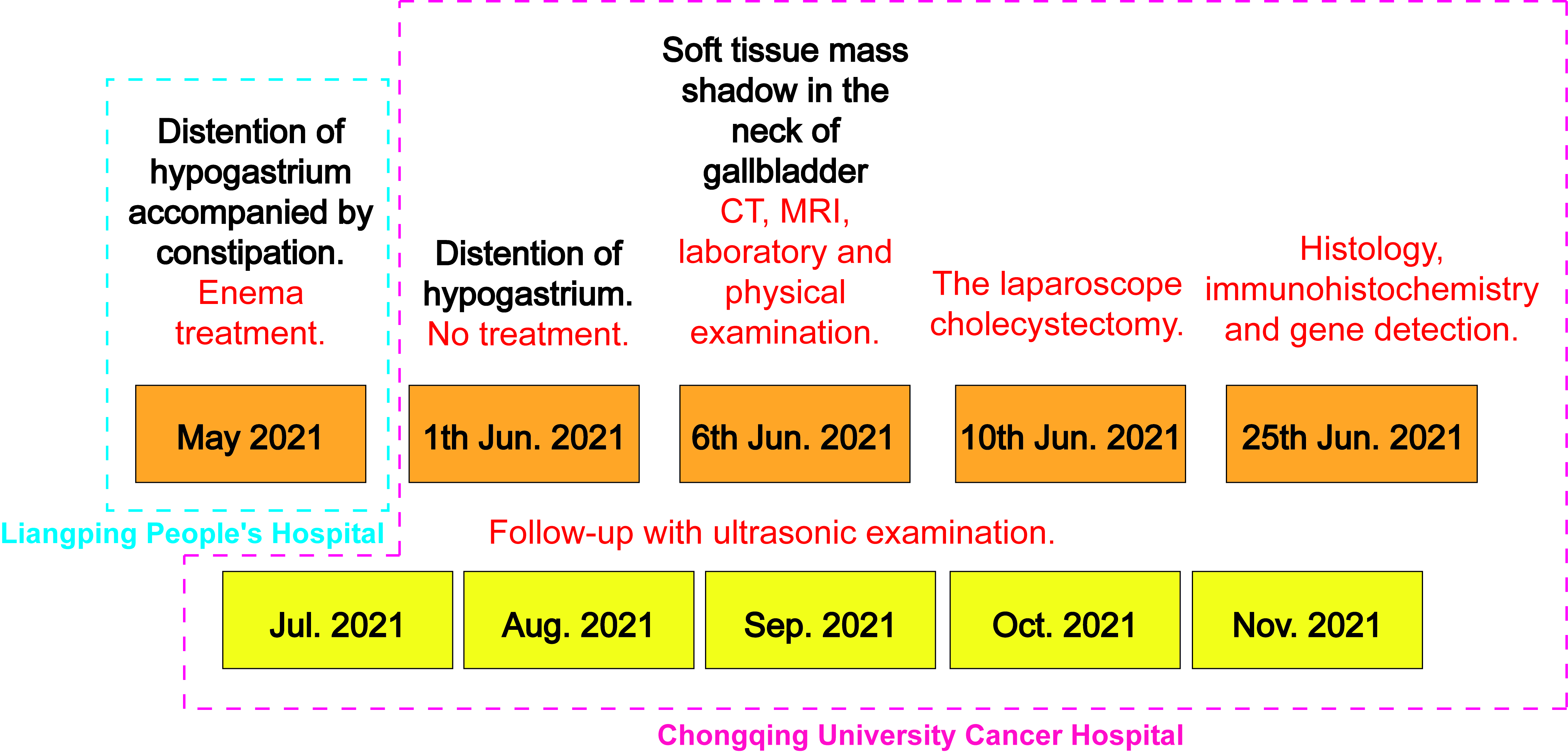

Supplement: Supplementary Figure 1 — Timeline with relevant data from the episode of care. [file Image_1.tif]
